# Switching of biological therapies in Brazilian patients with rheumatoid arthritis

**DOI:** 10.4155/fsoa-2018-0025

**Published:** 2018-12-04

**Authors:** Juliana Miranda de Lucena Valim, Fernanda Gomes Gonçalves Chaer, Fernanda D'Oliveira Guimarães da Silveira, Verônica Palmiro da Silva e Lima, Branca Dias Batista de Souza

**Affiliations:** 1 Médica assistente da Reumatologia da ISCMSP, Medicine Department/Rheumatology Division, Santa Casa de Misericórdia de São Paulo, São Paulo-SP/CEP 01221-900, Brazil; 2 Médica residente da Reumatologia da ISCMSP, Medicine Department/Rheumatology Division, Santa Casa de Misericórdia de São Paulo, São Paulo-SP/CEP 01221-900, Brazil

**Keywords:** anti-TNF, biologics, discontinuation, disease activity, remission rates, rheumatoid arthritis, switching

## Abstract

**Aim:**

To assess drug switching, rates of remission and disease activity in Brazilian patients with rheumatoid arthritis (RA) treated with biologic agents.

**Materials & methods:**

Using a retrospective method, a total of 94 adult patients were included.

**Results:**

Anti-TNF was the first choice therapy in 85 (90.4%) patients. After an average of 8 years of follow-up, 55 (59%) patients were taking anti-TNF, 18 (19%) abatacept, eight (9%) tocilizumab and 13 (14%) rituximab. In this period, 99 switches of biological therapy were registered in 55 patients.

**Conclusion:**

After 8 years of follow-up, 54% of the RA patients on biological therapy were still experiencing high or moderate activity despite established treatment, including switching between different biologic agents.

## Lay abstract

Biological therapies are new drugs made through biotechnology processes that have greatly improved the treatment of RA. However, sometimes biologic agents must be switched to another biologic or other therapy due to lack of response, intolerance, adverse effects or other reasons. This study aimed to assess drug switching, rates of remission and disease activity in Brazilian patients with RA treated with biologics. Within a follow-up of 8 years, 99 switches of biological therapy were registered in 55 patients. After 8 years of follow-up, activity of disease still remained high or moderate in nearly half of patients.

Rheumatoid arthritis (RA) is an autoimmune, chronic inflammatory disease characterized by joint swelling, joint tenderness and destruction of the synovial membrane of peripheral small and large joints [1–7].

The main characteristic of RA is the symmetrical polyarthritis, with a more frequent involvement of the hands and feet. The synovitis may be accompanied by extra-articular manifestations such as vasculitis, rheumatoid nodules, serositis, scleritis and interstitial lung disease.

RA affects approximately 0.5–1% of the population in Brazil. It is more prevalent in women (estimated female/male ratio of 2:1), and its incidence increases with age, showing a peak incidence around 30–50 years of age. RA reduces patient's quality of life and implies a severe burden to the patient, its family and the healthcare system.

In Brazil, considering a total population of about 207 million, and a prevalence of 0.46% for RA, it has been estimated that there is around one million people with the disease [5–8].

Treatment of RA comprises both pharmacological and nonpharmacological approaches. Treatment options include the use of medications, reduction of joint stress, physical and occupational therapy, and surgical intervention [4–7,11–18].

Treatment of RA has changed rapidly over the past decades, with therapies evolving to become increasingly specific, and aiming to neutralize inflammatory mediators implicated in the pathophysiology of disease. In the last decade, RA treatment has been significantly improved due to the introduction of biological disease-modifying antirheumatic drugs (DMARDs), which have demonstrated substantial benefits over conventional therapies. With the outstanding results of these target-specific therapies, RA treatment is no longer aimed only at improving symptoms, but at achieving disease remission. Currently, in Brazil, as in many other parts of the world, biological DMARDs are commonly used in patients with RA to decrease pain and inflammation, to reduce or prevent joint damage, and to preserve the structure and function of the joints [4,11–21].

Goal-based treatment can be defined as a ‘Treat to Target’ strategy aimed at achieving and maintaining remission, or at least a state of low disease activity [21]. To achieve this therapeutic goal in all patients, switches between different biological DMARDs have become frequent [19–26]. However, in Brazil, there is still little evidence about how to manage switches and which are the best sequences to follow when the therapeutic goal is not reached.

In Brazil, TNF inhibitors are the first choice among biologics when remission or low activity is not achieved with the use of synthetic DMARDs. When a failure of therapy with the initial biologic agent occurs or significant adverse effects appear, switching for another anti-TNF or another class of biologic drug may be considered [1–7,12–18].

Various biological DMARDs with different mechanism of action have been approved by Agência Nacional de Vigilância Sanitária, the Brazilian surveillance and regulatory agency, to be used in Brazil (Table 1), and switching among them is becoming an increasingly more common practice in Brazil, as in others parts of the world, in patients who have had an inadequate response or severe adverse events [1,2,5,18].

**Table 1.** **Biological disease-modifying antirheumatic drugs approved by Agência Nacional de Vigilância Sanitária in Brazil.**

**Mechanism of action**	**DMARDs**
TNF inhibitors	Adalimumab Certolizumab pegol Etanercept Golimumab Infliximab

B-cell depletion agent	Rituximab

T-cell costimulation inhibitor	Abatacept

IL-6 receptor antagonist	Tocilizumab

DMARD: Disease-modifying antirheumatic drugs.

Therefore, a retrospective study was conducted to assess drug switching in patients with RA treated with biologic agents following the guidelines of the Brazilian Society of Rheumatology and to describe the rates of remission or low activity disease according to DAS28 at the end of follow-up.

## Materials & methods

Using a retrospective design, we included patients from a rheumatology outpatient clinic that had sufficient information in the medical record to meet the inclusion criteria. Inclusion criteria were patients with RA, older than 18 years, with diagnostic data that met the ACR/EULAR 2010 criteria, and that were receiving regular treatment with biologic agents.

Our institution is a university hospital, and is a reference center in the treatment of RA in Brazil. All of our patients under biological therapy had visits every 3 months at least. Thus, we had new clinical data from these patients available in medical records every 3 months at least, since 2003, when we started the use of biologics.

About 450 patients with RA were followed in our institution over a period of 8 years, and 126 received biological therapy. Only patients with sufficient clinical information were included: 32 patients had insufficient data in medical records to be included or did not meet the inclusion criteria. Clinical information was collected from medical records twice a week, during 1 year, from May 2015 to May 2016.

Our institution follows the recommendations of the Brazilian Society of Rheumatology. Currently, the most recent recommendations for RA treatment is the ‘2012 Consensus of the Brazilian Society of Rheumatology for the treatment of rheumatoid arthritis’ [1]. Main recommendations of this Consensus state that immediately after diagnosis, a DMARD should be prescribed and treatment adjusted to achieve remission. Initial treatment should include synthetic DMARDs, and methotrexate is the drug of choice. Patients who fail to respond after two schedules of synthetic DMARDs should be evaluated for biological DMARDs, and only as an exception, the use of biologic DMARDs may be considered earlier. Anti-TNF agents are recommended as initial biologic therapy, and after therapeutic failure of a first biologic DMARD, other biologics can be used [1].

Demographics and clinical features were obtained from clinical records. Length of disease, presence of rheumatoid factor (RF) and bone erosion, medications already used, length of use of each medication, reasons for switching biologic agents and activity evaluation were collected from medical files. According to Disease Activity Score based on 28 joints (DAS28), patients were considered in remission with DAS28 ≤ 2.6 and a low activity disease was considered with a DAS28 value between 2.7 and 3.2.

Therapeutic failures to biological DMARDs were classified as: primary failure, when the patient did not reach therapeutic response 12 weeks after starting treatment (response was defined as a 0.6-point decline in DAS28); or secondary failure, when after an initial satisfactory response there was recurrence of the disease with increment of disease activity for more than 12 weeks.

A descriptive analysis of the drugs under study was performed using nonparametric methods of survival. Thus, it was possible to calculate the Kaplan–Meier estimates (median time to switch and probabilities of no switch in 1, 2 and 5 years) and compare the curves using the log–rank test. In order to compare the time to first switch between drugs, the Cox proportional hazards model was used [9]. This model calculates the hazard ratio (HR) that shows how great is the risk of an agent to be switched compared with another drug. Crude and adjusted models were analyzed (with control for age, sex, disease time, RF and erosive disease). All analytics and graphs were made using software R, version 3.4.1. [10]. For all analytics, a significance level of 5% was adopted.

Collected data were structured in a database using the Microsoft Excel 2013 program, and tables and graphs were also developed for data representation.

The study was approved by the Research Ethics Committee of Faculdade de Ciências Médicas da Santa Casa São Paulo (FCMSCSP), São Paulo, Brazil, with CAAE: 54696716.6.0000.5479.

## Results

Data were collected from 94 patients, of whom 84 (89.4%) were women. Mean age was 53 (±11.3) years and mean disease duration was 11.9 (±5.7) years; 75 (80%) patients had positive RF and 85 (91%) presented bone erosions at the time biological therapy was started (Table 2).

**Table 2.** **Clinical and epidemiological characteristics of 94 Brazilian patients with rheumatoid arthritis classified according to the first treatment with biologics.**

	**Total (n = 94)**	**ADA (n = 35)**	**ETA (n = 34)**	**INFL (n = 12)**	**GOL (n = 4)**	**ABATA (n = 5)**	**TOCI (n = 4)**
Age, mean (SD)	53 (±11.3)	53.9 (±10.3)	52.3 (±12.3)	49.9 (±10.2)	60.7 (±3.5)	50.2 (±15.9)	56.5 (±13.1)

Female, n (%)	84 (89)	33 (94)	29 (85)	10 (83)	4 (100)	4 (80)	4 (100)

Duration of disease in years, mean (SD)	11.9 (±5.7)	11 (±4.9)	10.9 (±4.3)	15 (±6.6)	15.7 (±6.7)	11 (±9.1)	17.2 (±8.5)

RF (positive), n (%)	75 (80)	29 (82)	27 (79)	8 (66)	3 (75)	4 (80)	4 (100)

Bone erosion, n (%)	86 (91)	29 (82)	33 (97)	11 (91)	4 (100)	5 (100)	4 (100)

ABATA: Abatacept; ADA: Adalimumab; ETA: Etanercept; GOL: Golimumab; INFL: Infliximab; RF: Rheumatoid factor; SD: Standard deviation; TOCI: Tocilizumab.

Before starting biological therapy, all patients were receiving conventional DMARDs: 64 (68%) were on methotrexate, 43 (46%) leflunomide, five (5%) antimalarial drugs, and one (1%) sulfasalazine. 38 were on combined cs-DMARD. The use of corticosteroids varied among the patients during the observation period, but at the beginning of the first biologic, 68 (72%) patients were receiving corticosteroids.

**Figure 1.**
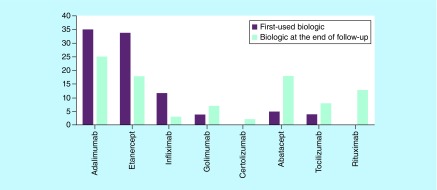
**Distribution of biological therapies used as first option and at the end of the 8-year follow-up in 94 rheumatoid arthritis patients.**

An anti-TNF was the first-choice therapy in 85 (90.4%) patients: 35 (37%) adalimumab, 34 (36%) etanercept, 12 (13%) infliximab and four (4%) golimumab. In five (5%) patients, the first choice was abatacept and in four (4%) tocilizumab. The mean follow-up of patients treated with biologics was 8 years. Ten patients were treated with biologic monotherapy over the observed period. At the end of follow-up, 59% were on anti-TNF (27% adalimumab, 19% etanercept, 3% infliximab, 7% golimumab and 2% certolizumab), 19% on abatacept, 9% on tocilizumab and 14% on rituximab (Figure 1).

**Figure 2.**
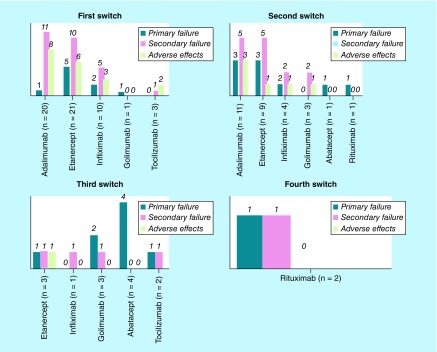
**Reasons for switching to biologics based on first switch and subsequent switching to another biologic drug.**

In an average period of 8 years, we observed 99 switches of biological therapy, 26% due to lack of initial efficacy (primary failure), 43% because of loss of efficacy over time (secondary failure) and 31% due to adverse effects (Figure 2).

**Figure 3.**
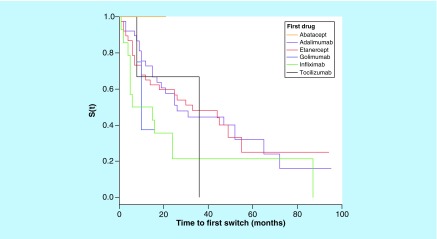
**Kaplan–Meier analysis of first biologic survival time until first switch.**

**Figure 4.**
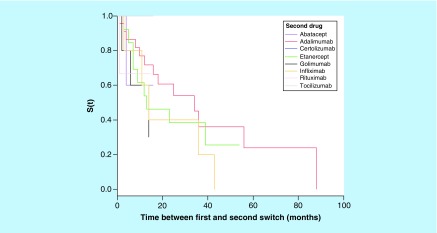
**Kaplan–Meier analysis of time between first and second switch.**

Drug survival rate was analyzed for each biologic using the time to first switch in patients with complete information regarding the start and end dates (Table 3 & Figure 3). The time between first and second switch was also calculated for each biologic (Table 4 & Figure 4).

**Table 3.** **Time to first switch analysis.**

**First drug**	**Number of patients^†^**	**Number of events (switch)**	**Median time (95% CI)**	**Prob. of no switch in 1 year**	**Prob. of no switch in 2 years**	**Prob. of no switch in 5 years**	**p-value^‡^**
Abatacept	5	0	–	–	–	–	0.49

Adalimumab	38	23	26 (17–NA)	0.73 (0.60–0.89)	0.58 (0.43–0.77)	0.32 (0.17–0.59)	0.49

Etanercept	38	23	33 (14–NA)	0.65 (0.51–0.82)	0.60 (0.46–0.78)	0.25 (0.11–0.55)	0.49

Golimumab	5	2	10 (8–NA)	0.37 (0.08–1)	–	–	0.49

Infliximab	14	12	10.5 (5–NA)	0.50 (0.30–0.84)	0.21 (0.08–0.58)	0.21 (0.08–0.58)	0.49

Tocilizumab	4	2	36 (8–NA)	0.67 (0.30–1)	0.67 (0.30–1)	0	0.49

^†^Number of patients with complete information regarding beginning and end dates.

^‡^Log-rank test.

CI: Confidence interval; NA: Not applicable.

**Table 4.** **Time between first and second switch analysis.**

**Second drug**	**Number of patients^†^**	**Number of events (switch)**	**Median time (95% CI)**	**Prob. of no switch in 1 year**	**Prob. of no switch in 2 years**	**Prob. of no switch in 5 years**	**p-value^‡^**
Abatacept	7	2	NA	0.60 (0.29–1)	–	–	0.72

Adalimumab	23	13	34 (16–NA)	0.72 (0.55–0.94)	0.61 (0.43–0.87)	0.24 (0.08–0.70)	0.72

Certolizumab	1	0	–	–	–	–	0.72

Etanercept	13	9	13 (7–NA)	0.54 (0.33–0.89)	0.38 (0.19–0.76)	–	0.72

Golimumab	5	3	14 (6–NA)	0.60 (0.29–1)	–	–	0.72

Infliximab	5	5	14 (11–NA)	0.60 (0.29–1)	0.40 (0.14–1)	0	0.72

Rituximab	3	1	NA	0.67 (0.30–1)	–	–	0.72

Tocilizumab	3	0	–	1	–	–	0.72

^†^Number of patients with complete information regarding beginning and end dates.

^‡^Log-rank test.

CI: Confidence interval; NA: Not applicable.

The Cox proportional hazards model was used to compare the time to first switch between drugs. The model calculates the HR that demonstrates the risk of a drug of being switched compared with another. Crude and adjusted models were analyzed applying controls for age, gender, disease time, RF (positive or negative) and erosive disease (Table 5).

**Table 5.** **Cox proportional hazards model. It shows the hazard ratio about the risk that a drug may be switched, compared with another agent.**

**Comparison**	**Crude models^†^**		**Adjusted model (n = 102)^†^**	

**Hazard ratio (95% CI)**	**p-value**	**Hazard ratio (95% CI)Hazard ratio (95% CI)**	**p-value**	
**Drug**				

Abatacept vs tocilizumab	–	–	–	–

Adalimumab vs tocilizumab	0.79 (0.18–3.38)	0.75	0.73 (0.17–3.21)	0.68

Etanercept vs tocilizumab	0.80 (0.19–3.43)	0.77	0.75 (0.17–3.38)	0.71

Golimumab vs tocilizumab	1.13 (0.16–8.07)	0.90	1.1 (0.15–8.04)	0.92

Infliximab vs tocilizumab	1.40 (0.31–6.34)	0.66	1.28 (0.28–5.95)	0.75

**Age**	0.99 (0.98–1.02)	0.94	1.00 (0.97–1.03)	0.98

**Gender**				

F vs M	1.01 (0.43–2.36)	0.98	0.74 (0.26–2.08)	0.56

**Disease time (years)**	1.01 (0.97–1.06)	0.68	1.00 (0.95–1.05)	0.98

**Rheumatoid factor**				

Positive vs negative	1.49 (0.76–2.93)	0.25	1.63 (0.74–3.59)	0.22

**Erosive**				

Yes vs no	1.10 (0.44–2.77)	0.83	1.04 (0.4–2.7)	0.94

^†^Cox regression.

CI: Confidence interval; F: Female; M: Male.

**Figure 5.**
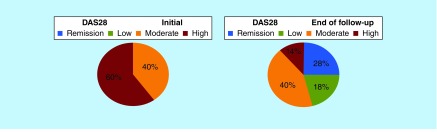
**Evaluation of disease activity prior to the use of biological therapy and at the end of the 8-year follow-up in 94 rheumatoid arthritis patients from Brazil.** Remission: ≤ 2.6; Low activity: 2.7 ≤ 3.2; Moderate activity: 3.3 ≤ 5.1; High activity: >5.1; DAS28-ESR was used for assessments. DAS28: Disease Activity Score based on 28 joints; ESR: Erythrocyte sedimentation rate.

At the start of biological therapy, 38 (40%) patients had moderate activity and 56 (60%) showed high disease activity. At the end of follow-up, after biologics switching as prescribed, we found 26 (28%) patients in remission, 17 (18%) had low activity, 38 (40%) moderate activity and 13 (14%) showed high disease activity (Figure 5).

**Figure 6.**
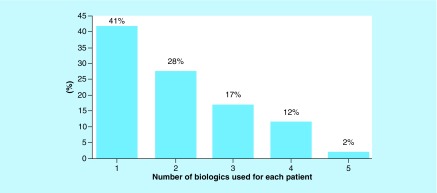
**Number of biological therapies used per patient during the 8-year follow-up.**

During the follow-up period, 39 (41%) patients remained on the first biologic, while 55 (59%) required at least one change of therapy. Of these, 26 (28%) patients had one switch and remained on the second biologic, 16 (17%) made two switches and were using a third biologic, 11 (12%) made three changes and were receiving the fourth biologic, and two (2%) patients made four switches and were using the fifth biologic, totaling 99 changes (Figure 6).

## Discussion

In the past two decades, major changes have occurred in the management of RA, including the definition of a therapeutic target and the application of a strict control with the necessary therapeutic changes and adaptations to achieve this goal. This concept is known as the ‘Treat to Target’ strategy, which is characterized by constantly seeking clinical remission or, at least, low disease activity. Recommendations for treating to target are nonspecific, since they do not advocate any particular type of intervention, but rather the necessary therapeutic changes whenever the target (remission or low disease activity) is not reached [12–16,19–26].

In the search for this therapeutic target, switching among biological therapies has become a common practice in patients with RA who have an inadequate response or unbearable adverse events. Therefore, physicians are increasingly familiar with the clinical profiles of different biologic agents and the evidence of their benefits in halting the progression of the disease [11–17].

Many authors reported that switching has become a common practice not only between anti-TNFs but also with alternative biologics that have different mechanisms of action [12–22]. In a survey conducted by Kamal *et al*. in the USA, the use of TNF inhibitors was assessed in 1970 rheumatologists. In this study, 94% of practicing rheumatologists reported switching patients from one anti-TNF to another if they had an inadequate response or intolerable adverse events [23].

Our study aimed to evaluate the need for switching of biologics in real life in a rheumatology service. We observed that anti-TNF drugs were the first option for biologic drugs after failure of synthetic DMARDs, which corresponded to 90% of our patients, as recommended by the Brazilian Consensus of Rheumatology (2012) for the treatment of RA [2,5]. In our series, 80% of patients who failed to respond to the first anti-TNF switched to another anti-TNF.

Several systematic and critical reviews suggest the benefits of cycling among TNF antagonists but all emphasize the need for prospective, randomized clinical trials to define the best treatment strategies for patients who cannot achieve or maintain an adequate response to an anti-TNF or are intolerant to that agent [11–16,24–26]. Although the evidence for therapeutic switching between TNF antagonists is limited, the results generally are in favor of this practice [14–19].

There are few controlled head-to-head studies to compare the results achieved with the switch between TNF antagonists [14-17]. Smolen *et al*. conducted a head-to-head comparative randomized 2-year study of certolizumab pegol versus adalimumab in 1488 RA patients (EXXELERATE study) [16]. Researchers aimed to compare the efficacy and safety of these two anti-TNFs and to assess the efficacy and safety of switching to the other TNF inhibitor without a washout period after insufficient primary response (primary failure) to the first anti-TNF at 12 weeks. In EXXELERATE, both anti-TNFs showed comparable responses at 3 months (12 weeks) and the number of patients with low disease activity was similar after 2 years of treatment (104 weeks). In this head-to-head comparison, no anti-TNF was shown to be superior compared with the other one. The study also demonstrated the clinical benefit and safety of switching to a second anti-TNF without a washout period after primary failure to a first TNF inhibitor [16].

There are no head-to-head studies to compare the results of switching between anti-TNFs and alternative biologics with a different mechanism of action. However, studies against placebo, such as REFLEX, conducted in patients who had failed at least one TNF antagonist, showed that rituximab, a B-lymphocyte depletion agent, provided a significantly greater improvement than placebo in the 24-week clinical results [27]. Another 6-month, randomized, controlled trial in RA patients with an inadequate response to anti-TNF therapy demonstrated superior efficacy with abatacept, a T-cell costimulatory inhibitor, against placebo [28]. Tocilizumab, an IL-6 receptor antagonist, was evaluated in a 24-week placebo-controlled randomized trial (RADIATE) in RA patients who demonstrated an inadequate response to one or more anti-TNF therapies and showed that the tocilizumab group had a better ACR20 response compared with the placebo group [29].

According to the Brazilian Registry for monitoring of biological therapies in rheumatic diseases, in a follow-up of 5 years, 32.4% of patients with rheumatologic diseases on biologic drugs discontinue therapy. Lack of effectiveness or loss of efficacy was the major reasons for discontinuation, accounting for 50% of patients who discontinued the treatment, followed by adverse events (27%) [18]. Findings from national registries, observational studies and clinical trials indicate that drug survival rates for TNF antagonists vary from approximately 65–83% at 1 year [11–15,22–26,30–32]. An analysis from an American cohort of 1549 patients with RA receiving anti-TNFs showed that within 1 year, 19% of patients discontinued or switched from biological therapies [19].

In a systematic research of articles that reported clinical outcomes of biologic treatment among RA patients with an inadequate response to TNF-α inhibitors, approximately 50 and 60% of primary and secondary failures, respectively, achieved an ACR20 response. In general, discontinuation rates were about 20–30% higher for secondary failures when compared with primary failures [20]. In our sample, 55 patients out of 94 (59%) discontinued the first biologic, secondary failure being the main reason, followed by primary failure and adverse effects.

A large North American cohort study evaluated the threshold in disease activity associated with switching biologic treatment regimens in RA patients in real-world clinical practice. These researchers found that the threshold to switch biologics decreased in the last years, especially among partial responders, and a disease state close or at low activity is considered ‘good enough’, by physicians and patients as well, to continue anti-TNF therapy [19]. Thus, if we consider that moderate activity according to DAS28 is a satisfactory target, our sample presents 86% of responses to treatment, which justify maintaining biologic therapy in patients with moderate activity.

In the 1-year analysis of a US cohort conducted by Zhang *et al*., the majority of patients (62%) were using the first anti-TNF, 30% the second and 8% the third. Those who maintained the first biologic presented lower disease activity according to Clinical Disease Activity Index (CDAI) compared with those that switched therapy (CDAI 8.4 vs 15.2, DAS28 3.1 vs 4.0). In addition, those who maintained the first biologic experienced a greater reduction in disease activity from the start of treatment compared with switchers (mean CDAI variation -7.7 vs -2.3; mean change in DAS28 -1.1 vs -0.3) [19]. In our series, a lower number of responses to therapy, based on DAS28, correlated to a greater number of biologics used by each patient.

In a systematic research conducted in 2011 by Rendas-Baum *et al*., the proportion of patients who achieved low disease activity (DAS28 < 3.2) and remission (DAS28 < 2.6) was lower among patients who had failed to respond to more than one anti-TNF previously than for patients failing to a single TNF inhibitor. This finding suggests that for patients with prior exposure to anti-TNF, the likelihood of a response to subsequent biologics declines with an increasing number of preceding anti-TNF therapies. However, studies analyzed in this systematic research differed in a number of factors, such as the type of biologics, length of disease and duration of treatment, which could have a significant influence on these estimates [20]. Gottenberg *et al*., in a study including 300 patients with RA that presented insufficient response to a first anti-TNF (persistent DAS ≥ 3.2), observed, after randomly switching to a second TNF or non-TNF biologic that more patients in the non-TNF group showed low disease activity compared with a second anti-TNF at 24 weeks (45 vs 28%; p = 0.004) and 52 weeks (41 vs 23%; p = 0.003). Therefore, according to these researchers, a non-TNF biologic was more effective in achieving a good or moderate disease activity than the second anti-TNF agent [26].

In our series, only 43 (46%) patients reached the therapeutic target of remission or low activity (28 and 18%, respectively) according to DAS28. This shows that despite the various possibilities of switching, there were still a large number of patients that had moderate (40%) or high disease activity (14%). Estimates derived from randomized clinical trials suggest that about 40–50% of RA patients treated for at least 6 months with one of the three first-generation TNF inhibitors (i.e., etanercept, adalimumab and infliximab) failed to achieve the ACR50 response criteria [30]. The results of a large study based on the Danish registry DANBIO indicated that more than 70% of patients with RA treated with adalimumab, etanercept or infliximab did not reach remission defined by DAS28 criteria [31]. Similarly, in the Swedish biologic registry Southern Sweden Antirheumatic Therapy Group (SSATG), remission was not achieved by 80% of patients after an average disease duration of more than 12 years. Overall, DAS28 remission and low disease activity were achieved in 20 and 43% of patients, respectively, which denote that 37% of patients were still suffering moderate or high disease activity [32].

## Conclusion

In our retrospective study, 99 biologics switches were assessed and the main reason for switching biological therapy was secondary failure. After an average of 8 years of follow-up, we found 28% of patients in remission and 18% of patients showing low disease activity according to DAS28. However, 54% of patients were still experiencing high or moderate activity despite established therapy, including switching between different biologic agents.

## Future perspective

Biological therapies had profoundly transformed the management of RA in the last two decades. Biologic agents directly target molecules and cells involved in the pathogenesis of RA and allow a better prognosis and clinical remission rate in patients with RA, especially in those who are not well controlled with traditional DMARDs. The future of biological products seems clearly promising with the greater understanding of the pathogenic mechanisms of RA and the constant advances in the area of biotechnology. In the coming years, new agents will be approved, improving the treatment and well-being of those patients who may not achieved a sustained remission with current agents.

**Summary points**

- At the end of an 8-year follow-up, 28% of patients were in remission, while 18% of patients had low disease activity.
- When moderate activity according to DAS28 is considered a satisfactory target, our sample demonstrated 86% of response to treatment, which justify maintaining biologic therapy in patients with moderate activity.
- In our sample, 59% of patients discontinued the first biologic. Secondary failure was registered as the main reason, followed by primary failure and adverse effects.
- In our series, 80% of patients who failed to respond to the first anti-TNF switched to another anti-TNF.
- After an average of 8 years of biological therapy, 54% of patients were still experiencing high or moderate activity despite established treatment.
